# *Pan*- and *core*- gene association networks: Integrative approaches to understanding biological regulation

**DOI:** 10.1371/journal.pone.0210481

**Published:** 2019-01-09

**Authors:** Warodom Wirojsirasak, Saowalak Kalapanulak, Treenut Saithong

**Affiliations:** 1 Systems Biology and Bioinformatics Research Group, Pilot Plant Development and Training Institute, King Mongkut’s University of Technology Thonburi (Bang Khun Thian), Bangkok, Thailand; 2 Bioinformatics and Systems Biology Program, School of Bioresources and Technology, King Mongkut’s University of Technology Thonburi (Bang Khun Thian), Bangkok, Thailand; Instituto Nacional de Medicina Genomica, MEXICO

## Abstract

The rapid increase in transcriptome data provides an opportunity to access the complex regulatory mechanisms in cellular systems through gene association network (GAN). Nonetheless, GANs derived from single datasets generally allow us to envisage only one side of the regulatory network, even under the particular condition of study. The circumstance is well demonstrated by inconsistent GANs of individual datasets proposed for similar experimental conditions, which always leads to ambiguous interpretation. Here, *pan*- and *core*-gene association networks (*pan*- and *core*-GANs), analogous to the *pan*- and *core*-genome concepts, are proposed to increase the power of inference through the integration of multiple, diverse datasets. The *core*-GAN represents the consensus associations of genes that were inferred from all individual networks. On the other hand, the *pan*-GAN represents the extensive gene-gene associations that occurred in each individual network. The *pan*- and *core*-GANs prospects were demonstrated based on three time series microarray datasets in leaves of *Arabidopsis thaliana* grown under diurnal conditions. We showed the overall performance of *pan*- and *core*-GANs was more robust to the number of data points in gene expression data compared to the GANs inferred from individual datasets. In addition, the incorporation of multiple data broadened our understanding of the biological regulatory system. While the *pan*-GAN enabled us to observe the landscape of gene association system, *core*-GAN highlighted the basic gene-associations in essence of the regulation regulating starch metabolism in leaves of *Arabidopsis*.

## Introduction

The accuracy and precision of inferring gene association networks (GANs) and data interpretation are dependent on the amount and quality of the underlying data, analytical methods employed and the experimental design. The integration of heterogeneous data and exhaustive utilization of all available information has become the frontier of biological research, especially in the post-genomic era. With the current advances in high-throughput technologies and the ever growing amount of genomic data, exemplified by the massive genome sequence data available in public databases, efficient and effective data utilization have become a major challenge. The concepts of *pan*- and *core*-genomes have been used to investigate the global and common gene sets in related species employing the huge amount of genome sequence data [1]. The concepts were originally introduced to integrate the genome information of bacteria [1] and have since been successfully applied to study eukaryotic organisms [2–5]. *Pan*-genome describes the union of nucleotide sequence entities (i.e. global set of genes) that exist in organisms within the same phylogenetic clade, and comprises the *core*-genome (essential nucleotide sequences shared by all genomes in the cohort), *dispensable* genome (nucleotide sequences shared by a subset of genomes in the cohort) and *strain-specific* genes (nucleotide sequences existing only within a particular genome in the cohort) [1, 6]. *Pan*-genomic approach has been widely employed to investigate genome diversity, pathogenesis and drug resistance, bacterial toxins and species evolution in bacteria [7–10], virus [11], fungus [2] and plant genomes [4, 5]. The contributions of these integrative data approaches, *i*.*e*. *pan*- and *core*- genomes, have been presented in a range of studies, and software packages and tools have been developed to facilitate their application [12].

The availability of transcriptome data has enabled the identification of genes differentially expressed under different conditions. Gene expression profiles provide the clue for decoding gene regulation and for identifying transcription factors and their associated target genes, mostly through the gene association network (GAN) [13]. The gene regulatory system is time and condition-specific, and this dynamism makes its assessment particularly challenging. The inference of dissimilar GANs from gene expression datasets that are largely comparable, with respect to experimental conditions, have been widely reported [14, 15], indicating a performance gap (accuracy and precision) and the need for improvement. Thus, the rationale to construct GAN by integrating multiple datasets, as against relying on consensus networks based on individual datasets, was proposed [14].

To pursue a novel conceptual analysis for transcriptome data integration and utilization, we constructed the *pan-* and *core-*gene association networks (*pan-* and *core-*GANs) employing multiple gene expression microarray datasets of *Arabidopsis thaliana* grown under diurnal conditions. The *core-*GAN, derived from the associated gene-pairs found in all employed datasets, represents the regulation that are related to the essential cellular processes, while the *pan-*GAN covers the entire gene-gene associations involved in the gene regulation, for the studied conditions. The performances of these gene association networks were evaluated and compared with those developed from individual datasets. Our results demonstrated the advantages of the *pan-* and *core-*GANs over the GANs from individual datasets.

## Materials and methods

### Data acquisition

Three time series microarray gene expression data of *Arabidopsis thaliana* grown under diurnal conditions, including Smith *et al*. (2004) [16], Blasing *et al*. (2005) [17] and Li *et al*. (2009) [18], were retrieved from the National Center for Biotechnology Information database (NCBI); the reference numbers of the experiments are GSE6174, GSE3416 and GSE11708, respectively (S1 Fig). The Affymetrix ATH-1 genome array platform contains approximately 22,000 *Arabidopsis* genes. The Smith *et al*.’s (2004) dataset is an eleven-point time series data (0, 1, 2, 4, 8, 12, 13, 14, 16, 20, 24 h) that describes gene expression in four-week-old *Arabidopsis* leaves during a 12 h diurnal cycle (12 h dark:12 h Light; 12D:12L); the six-point time series data (4, 8, 12, 16, 20, 24 h) by Blasing *et al*.’s (2005) describes gene expression in the leaves of a five-week-old *Arabidopsis* grown under 12 h diurnal (12L:12D) conditions; and Li *et al*. (2009) contains a five-point time series data (1, 4, 8.5, 12, 16 h) for 6-week-old *Arabidopsis* leaves during a short-day diurnal cycle (8L:16D).

The information on transcription factor (TF) genes of *Arabidopsis* was obtained from four databases including Plant Transcriptional Factor Database (PlantTFDB) version 3.0 [19], Database of *Arabidopsis* Transcription Factors (DATF) version 2.0 [20], *Arabidopsis* transcription factor database (AtTFDB) [21] and RIKEN *Arabidopsis* Transcription Factor database (RARTF) [22]. The families of regulator genes in the inferred networks were classified based on PlantTFDB, DATF, AtTFDB and RARTF databases.

The inferred gene association networks were evaluated against three reference networks of *Arabidopsis* to generalize our analysis and ensure that our results are not associated with a specific reference network. The reference networks included two co-expression networks that were based on 11,171 microarray gene chips and 328 RNA-seq datasets, obtained from ATTED database [23] and an experiment-based regulatory network obtained from the AtRegNet database [24]. The co-expression networks in ATTED database were inferred based on Pearson’s correlation coefficient (PCC). The reference network from AtRegNet database consisted of 10,193 genes with 16,109 interactions, whereas the microarray and RNA-seq based networks from the ATTED database consisted of 18,987 genes with 1,897,242 associations, and 19,708 genes with 1,865,890 associations, respectively.

### Gene association network inference

The gene association networks were inferred based on co-expression among the differentially expressed genes (DEGs) in each dataset. Herein, DEGs refer to genes whose patterns of expression differed across experimental conditions. They were, in practice, classified as the top five percentile of standard deviation (*sd*) for all expression profiles in the datasets. Then, the pair-wise relationships of genes were calculated based on the Pearson’s correlation coefficient, and only the gene pairs with |*PCC*| ≥ 0.9 and *p*-values ≤ 0.05 were included in the resulting gene association networks. Additionally, different cutoff criteria were also employed to ensure the validity of the results and conclusions. The *pan*- and *core*-GANs were constructed based on graphical integration of the union and interaction sets of the individual networks, respectively. The *core*-GAN represents the consensus associations of genes that were inferred from all individual networks. On the other hand, the *pan*-GAN represents the extensive gene-gene associations that occurred in each individual network.

### Network performance evaluation

The performances of the inferred gene association networks (GANs) were assessed using the network performance indices presented in Eqs 1–4:

$$\mathrm{accuracy}=\frac{\mathrm{TP}+\mathrm{TN}}{\mathrm{TP}+\mathrm{TN}+\mathrm{FP}+\mathrm{FN}},$$ (1)

$$\mathrm{precision}=\frac{\mathrm{TP}}{\mathrm{TP}+\mathrm{FP}},$$ (2)

$$\mathrm{specificity}=\frac{\mathrm{TN}}{\mathrm{TN}+\mathrm{FP}},$$ (3)

$$\mathrm{sensitivity}=\frac{\mathrm{TP}}{\mathrm{TP}+\mathrm{FN}}$$ (4)

where TP—true positive, FP—false positive, TN—true negative, and FN—false negative. All inferred networks were assessed using these performance indices on the basis of the given reference GAN of *Arabidopsis* derived from ATTED and AtRegNet databases.

## Results and discussion

### Inference of gene association networks based on three microarray datasets

The global gene expression data underwent reverse engineering analysis whereby the association of genes related to regulatory processes under prevailing conditions were inferred based on the gene co-expression hypothesis. The GANs that are based on individual datasets are often characterized by low precision usually caused by the temporal and spatial effects of the samples and the technical design such as replication and size of data series. In this section, we showed that under similar conditions, the GANs proposed to describe gene regulatory processes differed by the datasets employed in the co-expression analysis with respect to the network constituents, network performance and the biological insights conveyed by the networks. The study was conducted based on three largely comparable microarray time series datasets, including the eleven-point time series data by Smith *et al*., 2004 [16], the six-point time series data by Blasing *et al*., 2005 [17] and the five-point time series data by Li *et al*., 2009 [18] (S1 Fig).

#### Variation of network constituents

**Fig 1:**
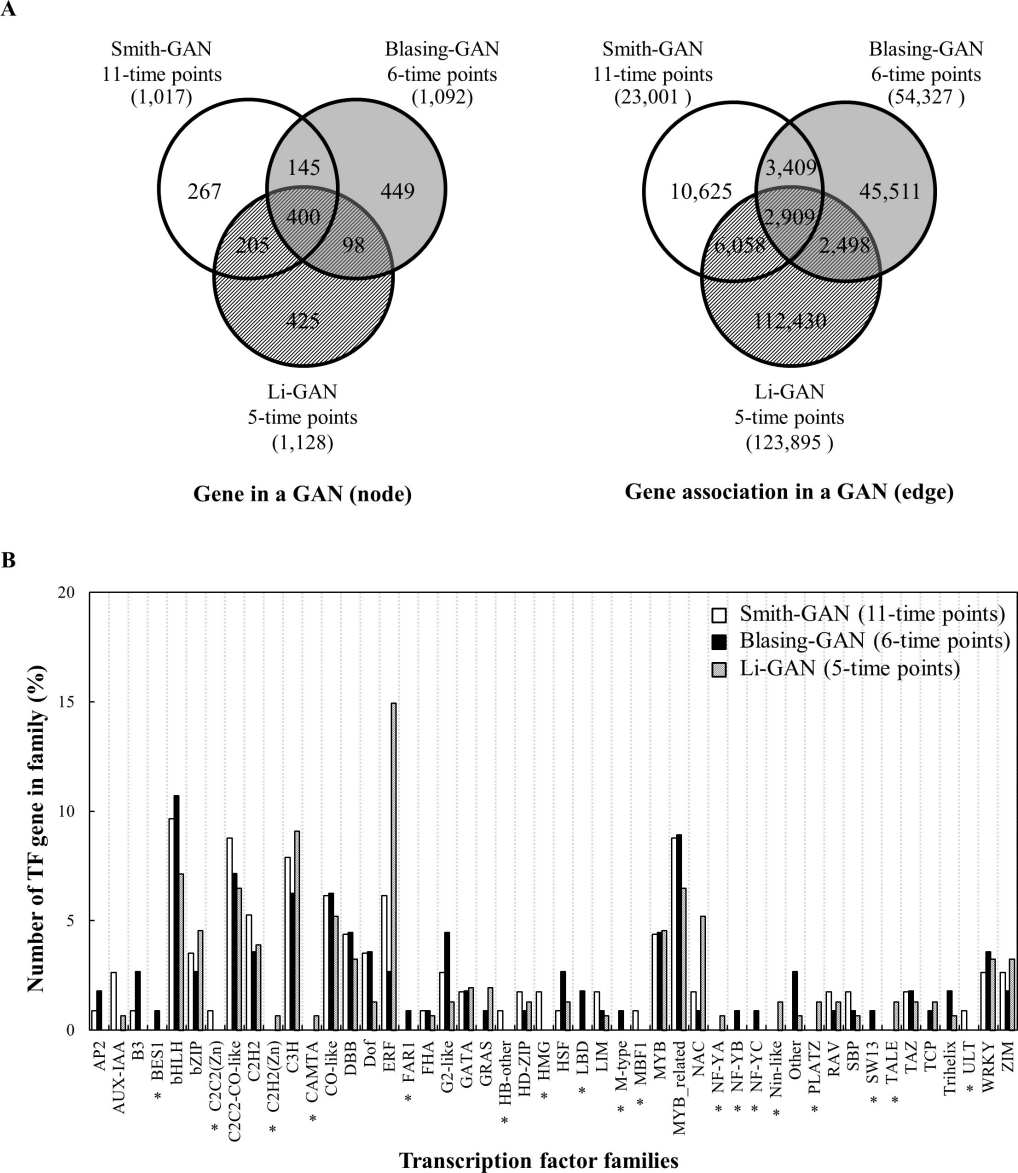
Variation of network constituents. The gene association networks, developed based on three gene expression data in leaves of *Arabidopsis* grown under diurnal conditions, were compared in terms of (A) the number of genes (left) and gene-associations (right), and (B) Percentage of number of TF genes in each transcription factor family, calculated from total TF genes in each GAN: Smith-GAN (114 TFs), Blasing-GAN (112 TFs) and Li-GAN (154 TFs). Black asterisks denote TF-families proposed by only one GAN.

We compared the three GANs developed based on the time series microarray data on gene expression in leaves of *Arabidopsis* grown under diurnal conditions (S1 Fig), hereafter referred to as Smith-GAN, Blasing-GAN and Li-GAN. The results demonstrated the diversity among the GANs in terms of network constituents, families of transcription regulators (TFs) and the TF-target gene associations. The Smith-GAN contains 1,017 genes (including 114 TFs) with 23,001 associations, Blasing-GAN contains 1,092 genes (including 112 TFs) with 54,327 associations and Li-GAN contains 1,128 genes (including 154 TFs) with 123,895 associations (Fig 1). The GANs differed significantly in relation to the number of gene associations, although they contain similar number of genes. Particularly, the Li-GAN, which was developed from a relatively low resolution dataset (five points), contains the highest number of genes and gene associations. This marked difference may be due to inherent effect of the data resolution on the resulting network [25, 26].

The three GANs were not only different in terms of the number of the genes and their associations, but also the biological information inferred from the functions of the constituent genes and transcription factors. Only 400 genes (~20% of all 1,989 genes in the three networks) and 2,909 gene-associations (~2% of all 183,440 associations in the three networks) were consistently proposed in all three GANs (Fig 1A). The GANs were also dissimilar with respect to the number of TF genes and the major TF-families proposed to be involved in the regulatory process, under the experimental conditions (Fig 1B). Each GAN contained at least 30 transcription factor families with different proportions of TF-families and TF genes; specifically, Smith-GAN contained 31 families, Blasing-GAN contained 36 families and Li-GAN contained 34 families. The major TF family found in Smith-GAN and Blasing-GAN was bHLH, which covered about 10 percent (11/114 and 12/112 genes, respectively) of the total TFs in the networks (Fig 1B). Despite sharing the major TF families, it was found that Smith-GAN contained five unique TF families including C2C2 (Zn), HB-other, HMG, MBF1 and ULT; and Blasing-GAN contained seven unique TF families including BES1, FAR1, LBD, M-type, NF-YB, NF-YC and SW13. Unlike the others, Li-GAN contained a large proportion of ERF TF-family, which accounted for about 15 percent (23/154) of the total TF genes in the network. Six unique TF families including C2H2 (Zn), CAMTA, NF-YA, Nin-like, PLATZ and TALE were found to be involved in transcriptional regulation.

The inferred GANs also differed in key transcription factors, which act as global regulators. The top 10 TFs, ranked on the basis of their connection with target genes in the network, were subsequently compared to examine the diversity of the GANs. None of the key TFs was found in all three GANs, and only three key TFs, including MYB-related TF (*At2g46830*), C2C2-CO-like TF (*At3g21890*) and DBB TF (*At2g21320*) were consistently inferred by Smith-GAN and Li-GAN. Ten unique key TFs were found in Blasing-GAN (S1 Table). These results highlighted the likelihood of overlooking key TFs involved in the regulatory mechanism when inferring GANs from individual datasets. For instance, the LBD TF (*At4g37540*) found in Blasing-GAN is involved in the regulation of many aspects of plant metabolism (*e*.*g*. controlling nitrogen (N) and nitrate (NO_3_^-^) uptake and assimilation in plant cells), growth and development [27]; and the Nin-like TF (*At2g43500*) found in Li-GAN is involved in the regulation of nitrate signaling during seed germination [28, 29].

#### Variation in network performance

**Fig 2:**
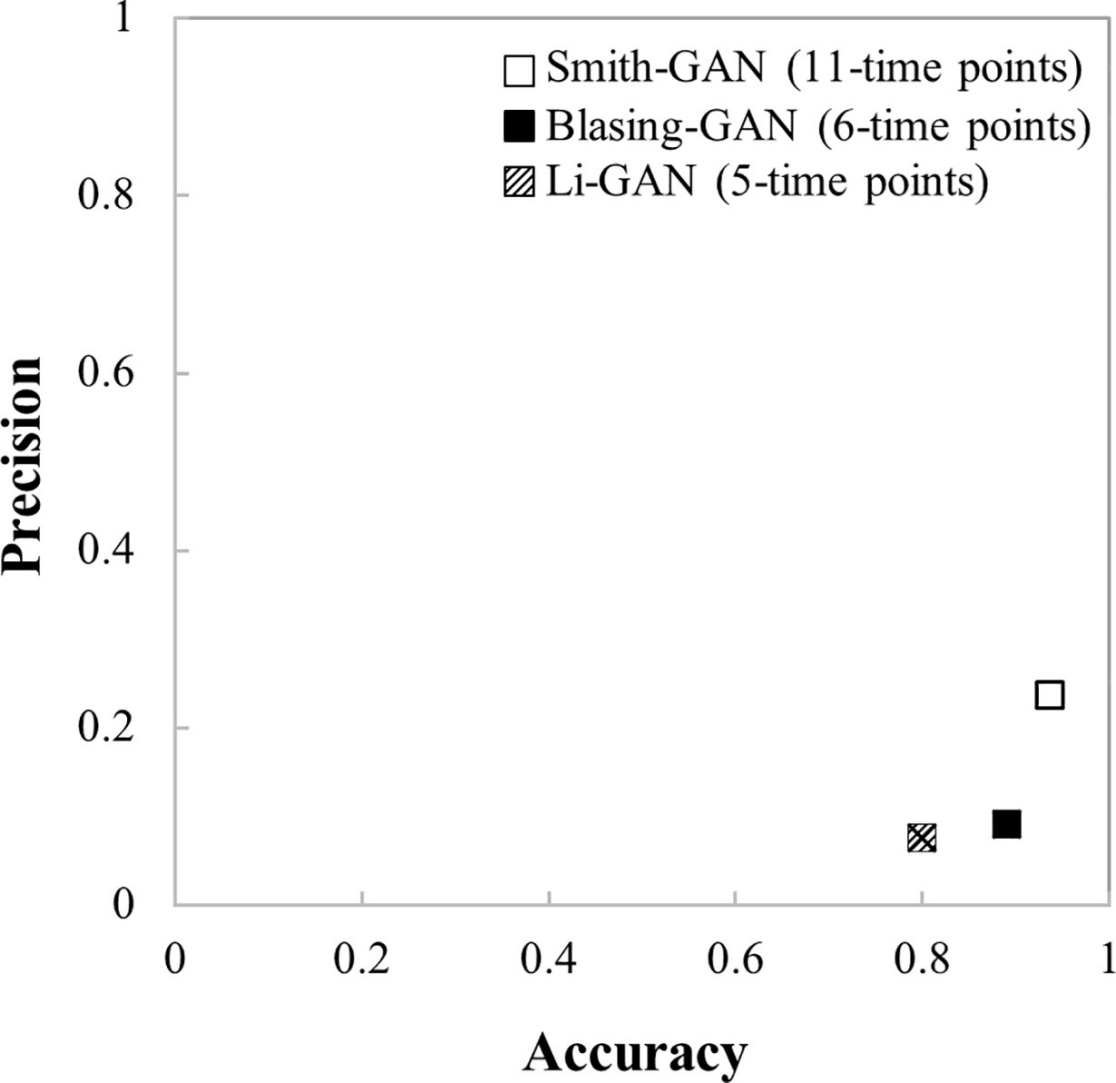
Performance of the inferred gene association networks (GANs). The performance of the three GANs of in leaves of *Arabidopsis* under diurnal condition was assessed based on accuracy and precision.

The performance of a GAN is influenced by the quality of data, method of data analysis and parameter settings employed [30]; and it is often assessed relying on indices such as accuracy and precision [25]. In this work, the performance of Smith-GAN, Blasing-GAN and Li-GAN was investigated based upon the reference co-expression network of *Arabidopsis* obtained from ATTED [23]. The accuracy and precision of the three inferred GANs differed markedly (Fig 2). Smith-GAN showed the highest accuracy (93.5%) and precision (23.7%), followed by Blasing-GAN (accuracy = 89.0% and precision = 9.1%), and Li-GAN (accuracy = 80.0% and precision = 7.6%) (S2 Table). These results corroborate previous findings that employing limited data increases the probability of false positive prediction, i.e. false identification of gene co-expression patterns with no biological relevance [31, 32], and highlight the predominant influence of the number of data points on transcriptional network inference. However, only 25% of the time series microarray data and RNA-seq in the Gene Expression Omnibus (GEO) database contain more than five data points [32], and there is a general lack of transcriptome data for higher eukaryotic organisms such as plants species. For cassava, in particular, only three transcriptome data on storage root development including Li *et al*. (2010) [33], Yang *et al*., (2011) [34] and Sojikul *et al*., (2015) [35] have been published till date, and they contain only 3–4 data points.

#### Variations in biological insights

**Fig 3:**
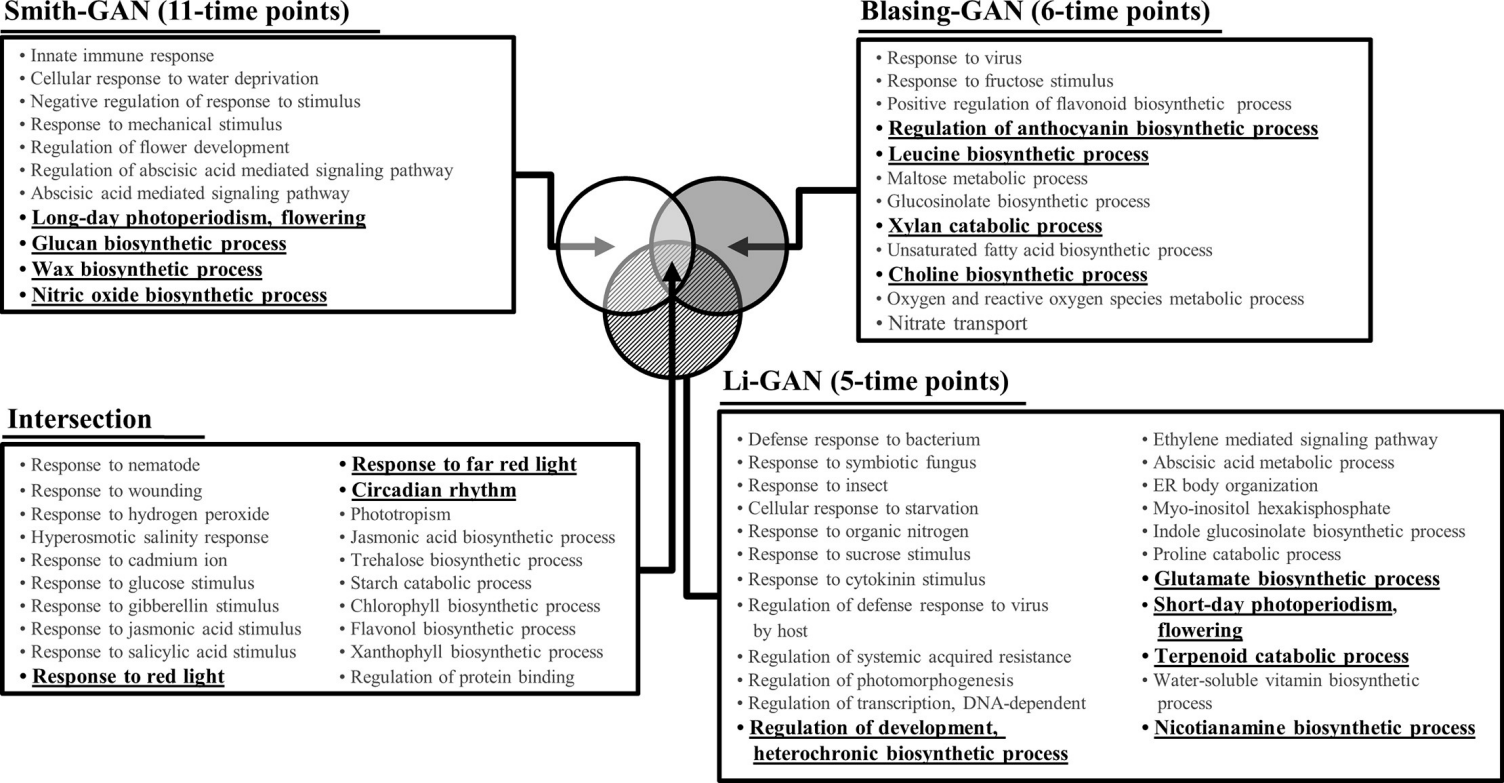
Variations in biological insights. The functional contents of the three gene association networks were investigated through gene ontology (GO) enrichment analysis. Biological functions significantly over-represented in the regulatory network were determined based on Bonferroni corrected *p-*value ≤ 0.05. The overlapped enriched GO terms exhibited the common predominant functions contained in the three GANs of *Arabidopsis* leaves under diurnal conditions.

In this section, we investigated the diversity of the GANs derived from individual datasets, regarding the biological content. The three GANs were subjected to gene ontology (GO) enrichment analysis, to determine the predominant biological processes involved in the regulatory network. The Smith-GAN was found to be enriched with 206 GO terms (535 genes with GO terms of total 1,017 genes), Blasing-GAN contained 162 GO terms (384 genes with GO terms of total 1,092 genes) and Li-GAN contained 248 GO terms (513 genes with GO terms of total 1,128 genes) (FDR ≤ 0.05). In total, 118 GO terms were found to overlap the three networks that may imply coincidence of the biological contents covered among GANs (Fig 3). The common GO terms are likely relevant to the transcriptional regulation of plants responses to stress, circadian rhythm and red/far-red light, which are key biological processes in *Arabidopsis* leaves under diurnal conditions [36, 37]

Although majority of the dominant biological functions of the inferred GANs were similar, the other half of the enriched GO terms varied with the employed datasets. These results shed light on the regulatory network, offering different perspectives based on the experimental design and measurement techniques employed, as demonstrated in Fig 3, but these are often ignored during analysis. The enriched GO terms, particularly those identified by the individual GANs, may additionally describe the regulatory processes occurring in the *Arabidopsis* leaves under diurnal conditions. The Smith-GAN is relevant to the wax biosynthetic process, glucan biosynthetic process, nitric oxide biosynthetic process, long-day photoperiodism and flower development. The Blasing-GAN and Li-GAN are more involved in the regulation of secondary metabolism such as choline biosynthetic process, xylan catabolic process, leucine biosynthetic process, anthocyanin biosynthetic process, terpenoid catabolic process, nicotianamine biosynthetic process, glutamate biosynthetic process, short-day photoperiodism and heterochrony.

### Integrated gene association networks for gene association study

Earlier, we showed how three time series microarray gene expression data in leaves of *Arabidopsis* grown under diurnal conditions were used to infer three GANs that are markedly different in many aspects, notwithstanding the largely comparable experimental conditions. To address the reliability concerns, efforts have been made in recent years to develop integrative approaches for inferring GANs. For example, the integration of gene associations inferred from several transcriptome datasets in a wide range of conditions [23] and the use of gene associations that are consistent across networks, based on meta data analysis and consensus analysis [14] have been proposed. The advantages and inherent drawbacks for these approaches have been debated and there is no best solution yet. In this work, we present alternative methods for studying GANs based on the integration of multiple datasets (*pan*-GAN and *core*-GAN) and show that both strategies might be essential for understanding the landscape of GANs and describing gene regulatory mechanisms. The *pan*-GAN and *core*-GAN approaches were used to exhaustively identify all possible gene sets and associations involved in cellular regulation, and infer the common gene-gene associations required for broad regulatory function, respectively.

**Fig 4:**
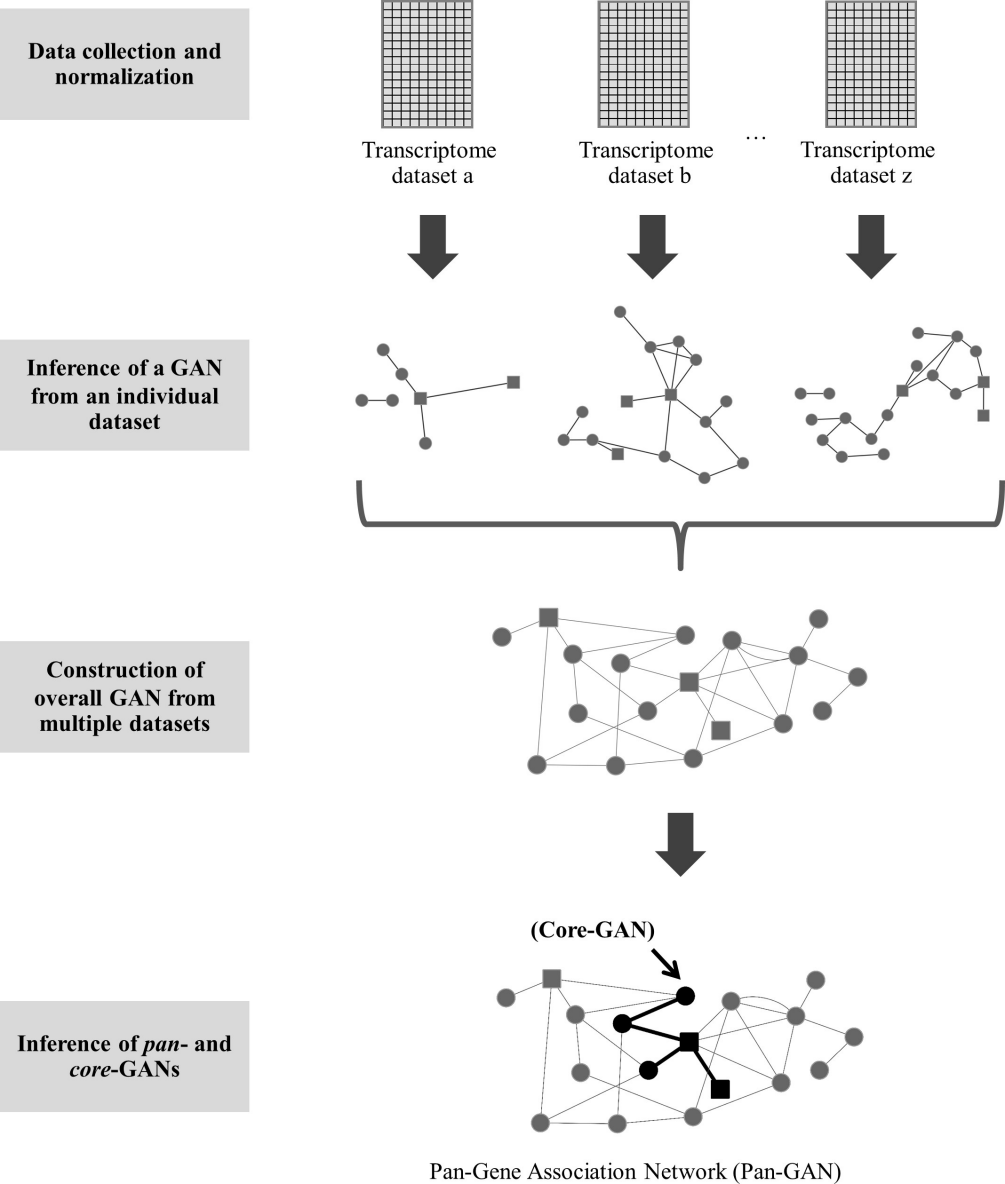
Conceptual framework of *pan-* and *core*-GANs for inferring transcriptional regulation using multiple transcriptome datasets. The framework presented in this work can be described in four steps: (1) data collection and normalization, (2) inference of GANs from individual datasets, (3) construction of overall GAN from multiple datasets and (4) inference of *pan*-GAN from all gene associations, and *core*-GAN from the consistent set of gene associations across individual networks.

The conceptual framework for developing *pan*- and *core*-GANs is illustrated in Fig 4. *Pan*-GAN combined all genes and gene associations that were inferred from the transcriptome datasets. Thus, it represents the overall genes and gene-pairs that might be involved in cell regulatory processes. The *core*-GAN, a subset of *pan*-GAN, was constructed based on a group of consistent network constituents (genes and gene-pairs). Besides the high-confidence prediction [15, 38, 39], *core*-GAN could employ common or primary regulatory machinery to manipulate normal cellular regulation. To examine this conceptual idea, integrated GANs were inferred from the three time series microarray datasets. The *pan*-GAN composed of 183,440 associations and 1,989 genes (including 235 TF genes), while *core*-GAN consisted of 2,909 associations and 321 genes (including 44 TF genes) (S2 Fig). Subsequently, the integrated networks were subjected to a performance analysis, as described earlier, and were compared with the GANs from individual datasets.

### Network performance of *pan*- and *core*-GANs

The performance of *pan*- and *core*-GANs was examined using the network performance indices consisting of accuracy, precision, specificity, sensitivity and false positive rate. For all inferred GANs (*i*.*e*. *pan*-GAN, *core*-GAN, Smith-GAN, Blasing-GAN, and Li-GAN), the validity of their gene associations was assessed against three independent reference networks including two co-expression networks developed from 11,171 microarray experiments and 328 RNA sequencing datasets deposited in ATTED database [23]; and the GAN of transcription factors (TFs) and their target genes (TGs) deposited in AtRegNet database [24]. The gene targets of TFs obtained from the AtRegNet database were identified based on: 1) the direct binding measurement of TF-TG, 2) the mutation experiments of TF-TG association in transgenic plants, and 3) the reported evidence of TF-TG regulation *in vivo*.

**Fig 5:**
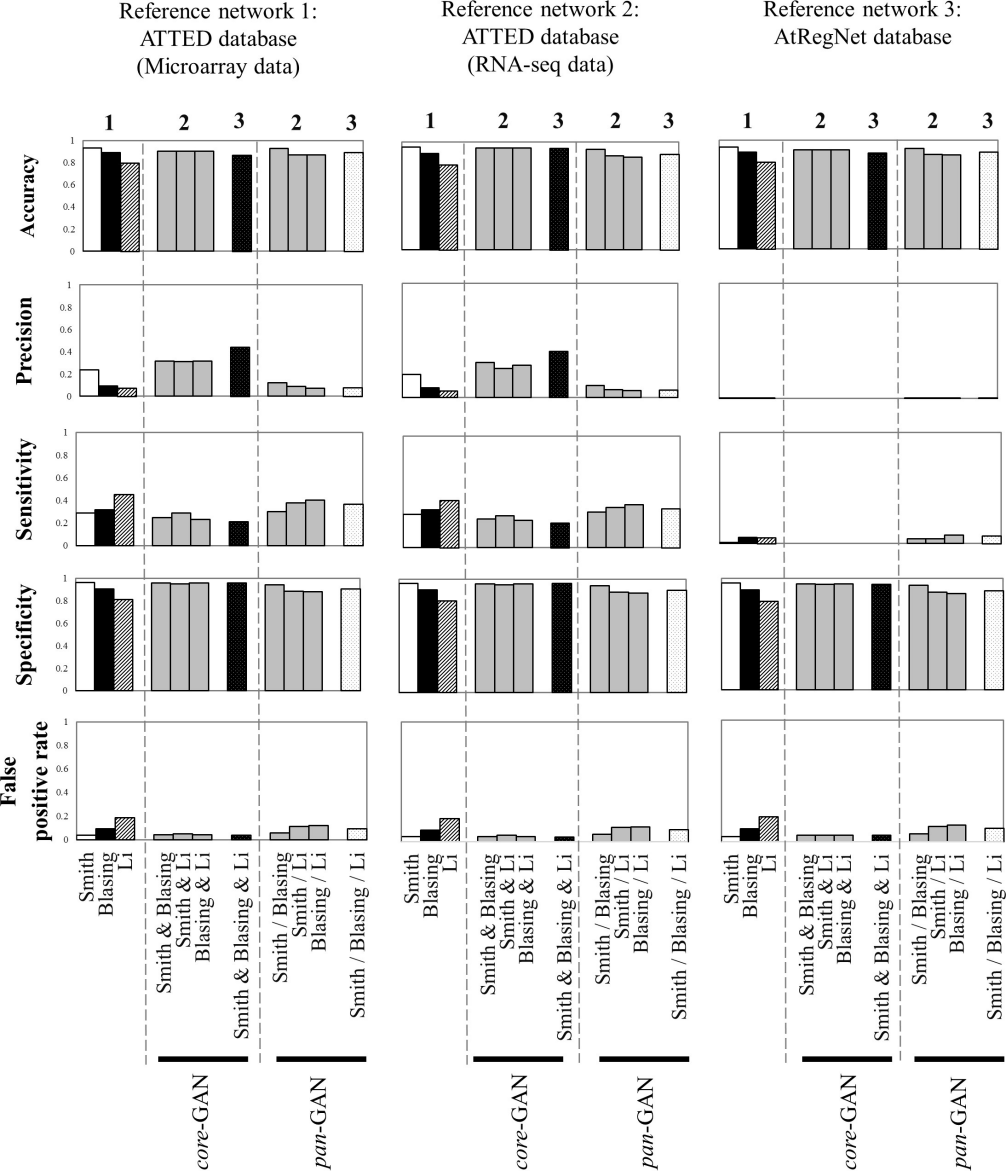
Comparison of the network performances of *core*-GAN, *pan*-GAN and the three GANs derived from individual datasets. The performance of all inferred networks was computed and compared with the three reference networks (two co-expression networks comprising 11,171 microarray datasets and 328 RNA-seq datasets from ATTED database, and one transcriptional regulatory network from AtRegNet database). The numbers (1 to 3) on the top of graphical column represent the number of transcriptome datasets from which the corresponding gene association network was inferred.

For the three reference networks, the analyses showed similar network performance and also gave the corresponding results of the comparative study between the GANs (*i*.*e*. GANs of individual datasets and GANs of the integrated datasets) (Fig 5 and S3 Fig). The performances of the GANs derived from individual microarray datasets were to a large extent dissimilar, but variations in the datasets only had a subtle effect on the overall performances of the integrated GANs. Among the GANs derived from single datasets, Smith-GAN, which was developed from a long time series dataset, performed best in almost all the measured indices, except sensitivity; and the opposite was the case for Li-GAN, derived from a short time series dataset. Smith-GAN exhibited about 93.5–95.5 percent accuracy, 95.7–96.2 percent specificity, 0–23.7 percent precision and 0.7–29.5 percent sensitivity; while Li-GAN was relatively poor in accuracy, specificity and precision, but had the highest sensitivity of up to 45 percent (S2–S4 Tables) when comparing among GANs inferred from individual datasets. These results highlighted the significance of data resolution (data point) on the transcriptional network inference, especially when using individual datasets. Long time series data (> eight data points) usually provide more defined expression patterns with distinct correlated and random profiles, which could help reduce the number of false positive predictions and improve the accuracy, precision and specificity of inferred networks.

Overall, the performance of the *pan*- and *core*-GANs was more robust to the number of data points (Fig 5). Integration of the datasets improved the performance of the inferred GAN especially when compared with Li-GAN derived from a short-series dataset. Fig 5 shows that *pan*-GAN and *core*-GAN derived from both two and three transcriptome datasets increased the accuracy and specificity levels of Li-GAN. The accuracy was increased from *c*.*a*. 80 percent to *c*.*a*. 93 percent (accuracy range of 86.1% - 93.4%) for *pan*-GAN, and *c*.*a*. 95 percent (accuracy range of 87.0% - 94.6%) for *core*-GAN. The specificity was improved from *c*.*a*. 80 percent to *c*.*a*. 94 percent (specificity range of 86.2%-94.3%) for *pan*-GAN and *c*.*a*. 96 percent (specificity range of 94.6–96.2%) for *core*-GAN. Furthermore, the level of false positive predictions in Li-GAN was reduced from *c*.*a*. 21 percent down to *c*.*a*. 6 percent (FPR range of 5.7% - 13.8%) in *pan*-GAN, and *c*.*a*. 4 percent (FPR range of 3.8% - 5.4%) in *core*-GAN. The comparative analysis of network performance showed corresponding results for all employed reference GANs in this study.

Despite the enhanced network performance, *core*-GAN resulted in the loss of valuable information that could be captured only in some datasets. The *core*-GAN rejected more than 70 percent of true positive interactions inferred by the analysis of the individual dataset (*e*.*g*. Smith-GAN: 3,702 interactions (76.0%), Blasing-GAN: 2,804 interactions (70.5%), Li-GAN: 6,632 interactions (85.0%)) (S2 Table). Compared with *pan*-GAN, 90 percent (10,768 interactions) of true positive interactions were abandoned in *core*-GAN or consensus-based network. These GANs served different purposes for example, the *core*-GAN offered a high-confidence network with better performance, while *pan*-GAN inferred extensive set of genes and associations that are probably involved in the transcriptional regulatory process.

### *Pan*- and *core*-GANs of starch metabolism in *Arabidopsis* leaves under diurnal conditions

**Fig 6:**
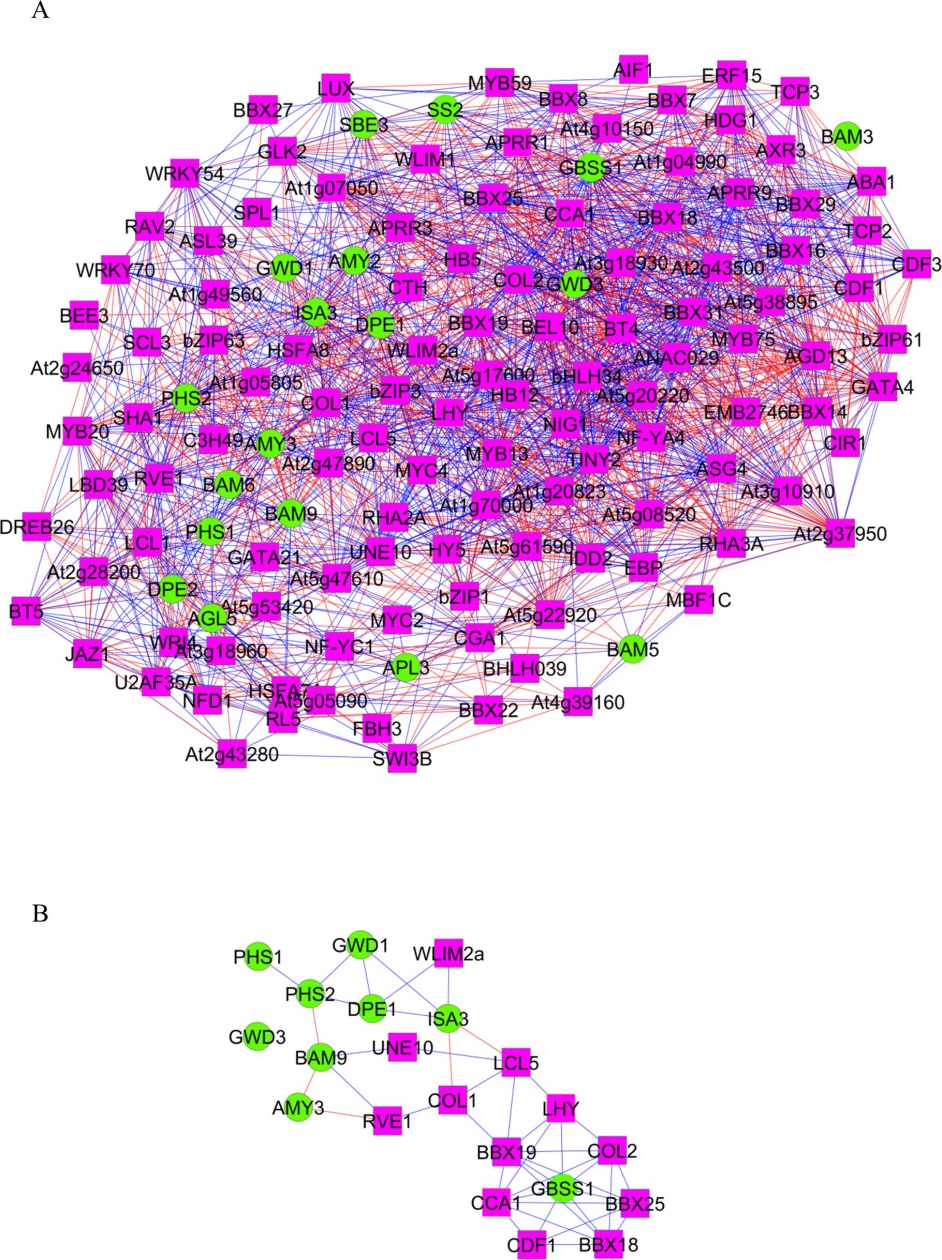
*Pan*- and *core*-GANs of starch metabolism in *Arabidopsis* leaves under diurnal conditions. Transcriptional regulation of starch metabolism was inferred by associations of starch metabolic genes and TF genes: (A) Starch-sub network based on *pan*-GAN and (B) starch-sub network based on *core*-GAN. The pink rectangles represent TF genes, the green circles represent starch metabolic genes, the blue and red lines represent positive and negative correlation between gene pairs, respectively.

To demonstrate the use of *pan*- and *core*-GANs in the inference of transcriptional regulation, GANs were constructed to investigate starch metabolism in leaves of *Arabidopsis thaliana* under diurnal conditions. The metabolism of starch in leaves is regulated by the synchronized rhythms of both diurnal and circadian cycles [16, 17, 40, 41]. The transcriptional regulation of starch metabolism in *Arabidopsis* leaves was studied through the network of gene association, focusing on the 48 starch-related genes suggested by Smith and colleagues [16]. The GANs developed based on the three microarray datasets covered, at most, only about 37 percent of genes (18 of 48 starch-related genes) related to the starch metabolism pathway. The *pan*-GAN contains 135 genes and 2,210 associations, and describes the transcriptional regulation of 18 starch metabolic genes (four genes of the synthesis pathway and 14 genes of the degradation pathway; Table 1) by 117 TF genes (Fig 6A). In contrast, *core*-GAN is substantially smaller and contains nine starch metabolic genes (one gene of synthesis pathway and eight genes of degradation pathway; Table 1), 12 TFs and 44 associations (Fig 6B). The results showed that transcriptional regulation of whole starch metabolism could not be observed, although multiple datasets were combined. Hence, it would be impossible to fully describe this regulation based on individual datasets.

**Table 1:** Starch metabolic genes presented in *core*-GAN and *pan*-GAN of *Arabidopsis* leaves under diurnal conditions.

AGI	Name	Description	*core*-GAN	*pan*-GAN
**Starch biosynthesis**				
*At4g24620*	PGI1	Phosphoglucoisomerase		
*At5g51820*	PGM1	Phosphoglucomutase		
*At5g19220*	APL1	ADP glucose pyrophosphorylase large subunit 1		
*At1g27680*	APL2	ADP glucose pyrophosphorylase large subunit 2		
*At4g39210*	APL3	ADP glucose pyrophosphorylase large subunit 3		√
*At2g21590*	APL4	ADP glucose pyrophosphorylase large subunit 4		
*At5g48300*	APS1	ADP glucose pyrophosphorylase small subunit		
*At1g05610*	APS2	ADP glucose pyrophosphorylase small subunit-like		
*At5g24300*	SS1	Starch synthase I		
*At3g01180*	SS2	Starch synthase II		√
*At1g11720*	SS3	Starch synthase III		
*At4g18240*	SS4	Starch synthase IV		
*At1g32900*	GBSS1	Granule-bound starch synthase	√	√
*At3g20440*	SBE1	Starch branching enzyme I		
*At5g03650*	SBE2	Starch branching enzyme II		
*At2g36390*	SBE3	Starch branching enzyme III		√
				
**Starch degradation**				
*At5g65685*	GLS1	Glucan synthase-like		
*At2g39930*	ISA1	Starch debranching enzyme: Isoamylase I		
*At1g03310*	ISA2	Starch debranching enzyme: Isoamylase II		
*At4g09020*	ISA3	Starch debranching enzyme: Isoamylase III	√	√
*At5g04360*	LDA1	Starch debranching enzyme: Limit dextrinase		
*At1g10760*	GWD1	Glucan water dikinase 1	√	√
*At4g24450*	GWD2	Glucan water dikinase-like 2		
*At5g26570*	GWD3	Glucan water dikinase-like 3	√	√
*At5g64860*	DPE1	Glucanotransferase	√	√
*At2g40840*	DPE2	Transglucosidase		√
*At3g29320*	PHS1	Glucan phosphorylase (plastidial)	√	√
*At3g46970*	PHS2	Glucan phosphorylase (cytosolic)	√	√
*At4g25000*	AMY1	a-Amylase 1		
*At1g76130*	AMY2	a-Amylase 2		√
*At1g69830*	AMY3	a-Amylase 3	√	√
*At3g23920*	BAM1	b-Amylase 1		
*At4g00490*	BAM2	b-Amylase 2		
*At4g17090*	BAM3	b-Amylase 3		√
*At5g55700*	BAM4	b-Amylase 4		
*At4g15210*	BAM5	b-Amylase 5		√
*At2g32290*	BAM6	b-Amylase 6		√
*At2g45880*	BAM7	b-Amylase 7		
*At5g45300*	BAM8	b-Amylase 8		
*At5g18670*	BAM9	b-Amylase 9	√	√
*At3g23640*	AGL1	a-Glucosidase-like 1		
*At5g63840*	AGL2	a-Glucosidase-like 2		
*At3g45940*	AGL3	a-Glucosidase-like 3		
*At5g11720*	AGL4	a-Glucosidase-like 4		
*At1g68560*	AGL5	a-Glucosidase-like 5		√
*At5g46110*	TPT1	Triose phosphate translocator		
*At5g16150*	GLT1	Glucose transporter		
*At5g17520*	MEX1	Maltose exporter		

**Fig 7:**
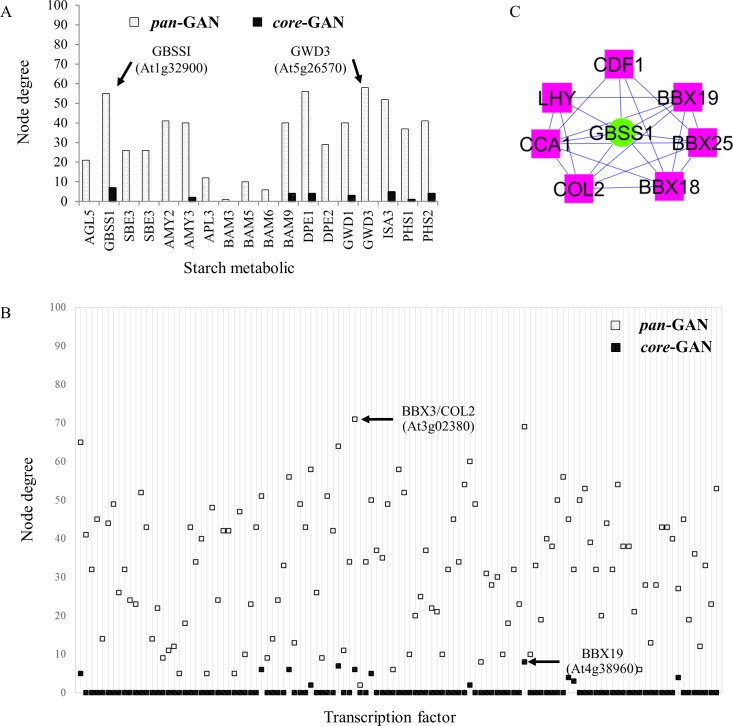
Node degree of genes in starch-sub network of *core*-GAN and *pan*-GAN of *Arabidopsis* leaves under diurnal conditions. (A) Node degree of starch metabolic genes, (B) node degree of transcription factor, and (C) the potential transcriptional regulators of *GBSSI* gene based on *core*-GAN. The pink rectangles represent TF genes, the green circles represent starch metabolic genes, the blue and red lines represent positive and negative correlation between gene pairs, respectively.

The consensus-based network, proposed herein as *core*-GAN, is generally considered a reliable network because the constituents are supported by more than one independent study, making it a primary network that represents the basic transcriptional regulatory process of the system. Accordingly, our proposed *core*-GAN was exploited in the identification of the important genes that play a major role in starch metabolism under diurnal cycle. These genes were basically defined by the number of associations (*i*.*e*., node degree); highly associated genes were denoted as hub genes. The degree of association reflects the influence of such a gene on the overall regulatory network. It suggests the tightly regulated genes for a target-gene hub and the global regulator for a TF-gene hub. Through graphical analysis, node degree of all genes in GANs of starch metabolism was determined and shown in Fig 7. Among the nine starch metabolic genes in *core*-GAN, *Granule-Bound Starch Synthase* (*GBSS*: *At1g32900*) had the highest node degree (= seven; called a hub gene) and was found to be associated with seven neighbors (Fig 7A). The result corroborates the reported significant role of *GBSS* in amylose and starch biosynthesis [42]. Depletion of the *GBSS* function crucially affects amylose content in starch granules of plant species such as *Arabidopsis* [43, 44], sweet potato [45], cassava [46] and wheat [47]. Regarding the transcription factor genes, *B-Box Domain Protein 19* (*BBX19*: *At4g38960*) was identified as a hub by a node degree of eight (Fig 7B). *BBX 19* is reported to be a key regulator involved in the growth and developmental processes, including seedling photo-morphogenesis [48] and regulation of photoperiodic flowering [49].

Furthermore, the gene co-expression network could suggest the mechanism underlying the influence of diurnal cycle on starch metabolism. The proposed *core*-GAN showed that starch metabolism was tightly regulated by the endogenous circadian clock which allowed the intracellular process of plant to be entrained by the diurnal cycle. As demonstrated in the sub-network of *core-*GANs, *GBSS* was found to be regulated by seven transcription factors under diurnal conditions based on the co-expression hypothesis; the transcription factor families included four zinc-finger (*BBX3/COL2*: *At3g02380*, *BBX18*: *At2g21320*, *BBX19*: *At4g38960* and *BBX25*: *At2g31380*) [50], two MYB (*CCA1*: *At2g46830* and *LHY*: *At1g01060*) and one Dof (*CDF1*: *At5g62430*) (Fig 7C). Correspondingly, it has been reported that the expression of *GBSS* gene might be regulated by the core circadian clock TFs, *CCA1* and *LHY* [51]. Also, the *CCA1* and *LHY* genes are the main regulators for *BBX18*, *BBX19* and *BBX25* [52], and the co-expression profiles under constant light condition suggest they also regulate *BBX3* [53–55].

In addition to the co-expression evidence, the regulation of *GBSS* by *CCA1* and *LHY* genes was also supported by the existing circadian clock-specific transcriptional binding site on the promoter. It was reported that upstream promoter of *GBSS* gene in *Arabidopsis* contains *cis*-regulatory element (AACAAATCT) for CCA1 TF binding [51]. However, a phylogenetic study of *GBSS* genes in monocots and eudicots revealed the genomic structure of *GBSS* genes are largely similar within the same plant cohort, but distinct across cohort [56]. Thus, transcript abundance of *GBSS* might be controlled by different regulators. The expression of *GBSS* gene in leaves of *Arabidopsis* is regulated by the circadian clock of CCA1 and LHY proteins [51], whereas in rice endosperm, it is controlled by two interacting proteins of the MYC and EREBP families [57].

In contrast with the *core*-GAN, which relied on high precision data and low network coverage, *pan*-GAN provided the extensive gene regulatory network with considerably good overall performance (Fig 5 and S2–S4 Tables). It illustrated the atlas of the transcriptional regulatory process for starch metabolism in *Arabidopsis* leaves during light/dark cycles which covered all correlated genes identified in the individual datasets (Table 1). According to *pan*-GAN of starch metabolism, *GBSS* was also highly regulated by genes in starch biosynthesis pathway with the largest set of correlated genes (55 neighbor genes), while *GWD3* was identified for starch degradation pathway in the same manner (58 neighbor genes) (Fig 7A). For the transcription factor, *BBX3*/*COL2* was identified as the hub regulator for this GAN with 71 correlated genes (Fig 7B). The large coverage of *pan-*GAN could help certify the hub potential of highly regulated genes identified from the confined set of *core-*GAN. *Pan*-GAN, in addition, enabled us to envisage the global view of the gen regulatory network for the studied system that could not be well inferred by *core-*GAN.

**Fig 8:**
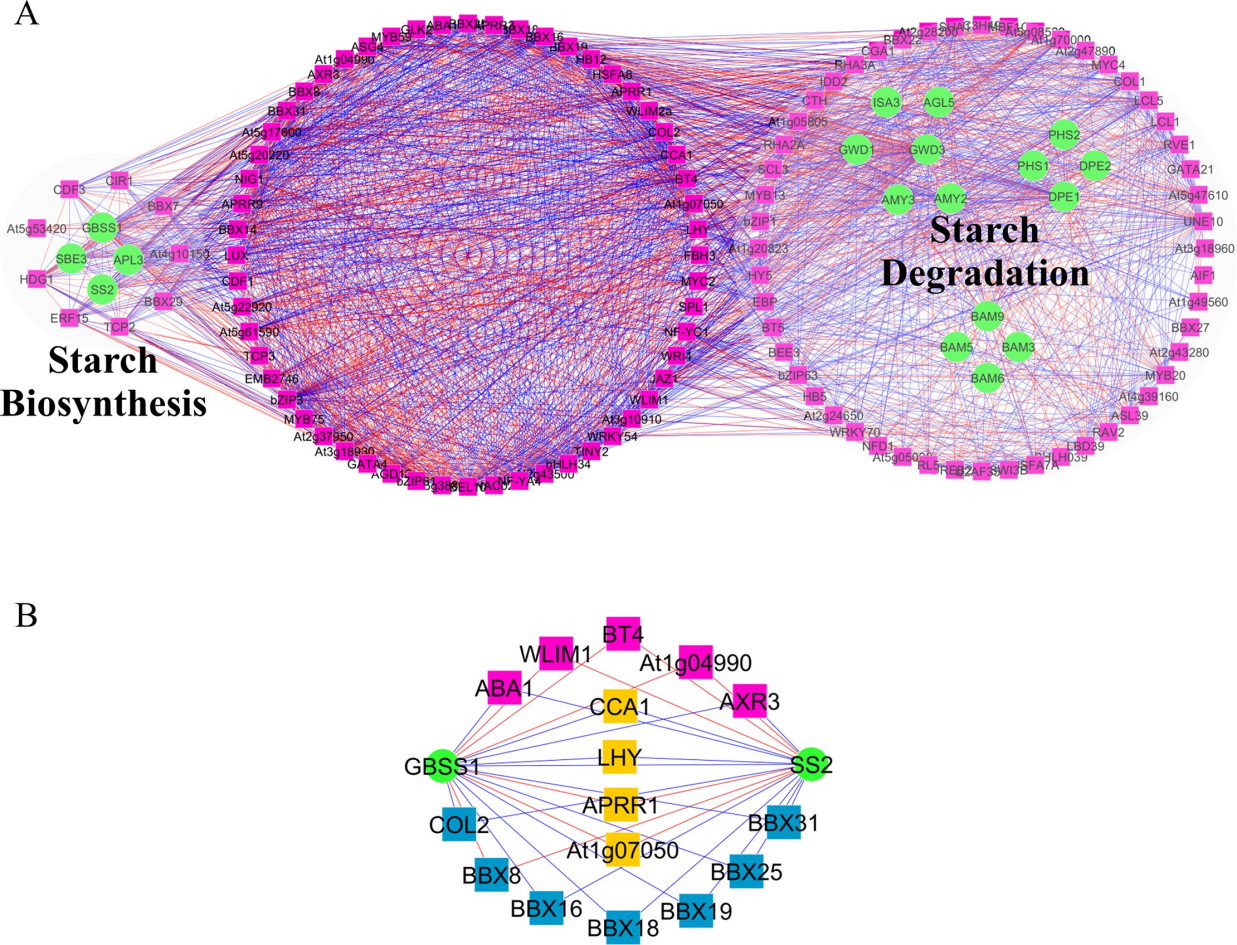
*Pan*-GAN for exploring the gene regulation underlying starch metabolism in leaves of *Arabidopsis* under diurnal conditions. (A) Gene association network demonstrating the role of TF genes in the regulation of starch biosynthesis and degradation in *Arabidopsis* leaves (B) A group of TF genes co-regulating *GBSSI* and *SS2* genes; green circles—starch genes, orange rectangles—circadian clock-related TF genes, blue rectangles—zinc-finger B-box TF gene, pink rectangles—other TF families, blue lines—positive correlation between gene pairs, and red lines—negative correlation between gene pairs.

The *pan*-GAN of the starch metabolism explicitly showed that starch synthesis and starch degradation pathways were tightly regulated by the same set of transcriptional regulators under diurnal conditions (Fig 8A). The results indicated that up to 48 percent of the transcription factor genes (56 of 117) related to starch metabolism likely regulate both the starch synthesis and degradation pathways. For instance, *pan-*GAN suggested that *BBX3* (*COL2*), *CCA1* and *LHY* transcription factors were the regulators of two starch biosynthesis gene (positive correlation: *GBSS* and *SS2* genes) and five starch degradation genes (negative correlation: *GWD1*, *GWD3*, *AMY3*, *ISA3* and *DPE1* genes), yet in an antagonistic manner. Another 61 TFs were found to be related with either starch synthesis genes (nine TFs) or starch degradation genes (52 TFs).

Approximately 75 percent of TFs in *pan*-GAN regulating starch metabolism (pink squares in S4 Fig) were associated with circadian-genes (orange diamond in S4 Fig), including *CCA1*, *ELF3*, *ELF4*, *GI*, *LHY*, *LUX*, *PRR3*, *PRR9* and *TOC1* [37]. *Pan*-GAN revealed that 16 TFs cooperatively control *GBSS1* and *SS2* genes (Fig 8B), and 11 of the TFs are circadian-related regulators (central clock genes: *LHY*, *CCA1*, *APRR1* and *At1g07050;* and 7 BBX TF genes) that could not be captured by *core*-GAN. These observations supported the coordination between *GBSS1* and *SS2* which affects starch composition.

## Conclusions

In this study, *pan*- and *core*-gene association networks (*pan*-GAN and *core*-GAN) are proposed to improve our understanding of the biological regulatory system and address the issues network reliability and sensitivity to data quality, often associated with GANs inferred from individual datasets. Overall, enhanced network performance was achieved by incorporating multiple transcriptome dataset into a single network (Fig 4). Overall, the *pan*- and *core*-GANs performed better than GANs derived from individual datasets, and they were also more robust. The *pan*-GAN captured all gene sets and associations involved in cellular, totaling 1,989 genes, 183,440 associations and 235 TF genes. The *core*-GAN consisted of 2,909 associations, 321 genes and 44 TF genes (S2 Fig), representing the basic gene-gene associations, common in all datasets employed, required for broad regulatory function. These integrative approaches are promising tools for improving our understanding of the gene regulatory processes.

## Supporting information

S1 Fig The conditions of time-series microarray datasets. (1) Smith *et al*. (2004) collected the data at 1, 2, 4, 8 and 12 hours during the dark, and light periods (2) Blasing *et al*. (2005) collected the data at 4, 8 and 12 hours in both light/dark cycle conditions and (3) Li *et al*. (2008) collected the data at 1 and 4 hours during the light period and at 0.5, 4 and 8 hours during the dark period.
(TIF)

S2 Fig *Pan*- and *core-* gene association networks of *Arabidopsis* leaves under diurnal condition. (A) *pan*-gene association network (*pan*-GAN) and (B) *core*-gene association network (*core*-GAN). The pink rectangles represent transcription factor genes, and the orange diamonds represent other genes (*i*.*e*., metabolic genes and signaling proteins). The gray symbols represent genes of *pan*-GAN that were absent in the *core*-GAN. The red and blue lines denote negative and positive correlation, respectively.
(TIF)

S3 Fig Comparison of the network performances of *core*-GAN, *pan*-GAN and the three GANs derived from individual datasets whereby the GANs were developed based on different cutoff criteria of correlation coefficient. (A) cut-off varied according to the absolute magnitude of PCC values; (B) cut-off varied according to relative percentile rank of PCC values.
(PDF)

S4 Fig *Pan*-GAN for inferring the regulation of starch metabolic genes by circadian clock. The orange diamonds represent circadian clock-related genes, the pink rectangles and the green circles represent TF and starch genes that are related to circadian clock genes. The gray symbols represent genes that are not correlated with circadian clock-related genes. The red and blue lines denote negative and positive correlation, respectively.
(TIF)

S1 Table Top ten TF genes (hub genes) in the GANs inferred from individual gene expression datasets, i.e. Smith-GAN, Blasing-GAN and Li-GAN. (PDF)

S2 Table Comparing the performance of Smith-GAN, Blasing-GAN, Li-GAN, *core*-GAN and *pan*-GAN using the co-expression network from 11,171 microarray datasets (ATTED database) as a reference network. (PDF)

S3 Table Comparing the performance of Smith-GAN, Blasing-GAN, Li-GAN, core-GAN and pan-GAN using the co-expression network from 328 RNA-seq datasets (ATTED database) as a reference network. (PDF)

S4 Table Comparing the performance of Smith-GAN, Blasing-GAN, Li-GAN, *core*-GAN and *pan*-GAN using the transcriptional regulatory network, based on direct TF-TG interactions, in AtRegNet database as a reference network. (PDF)
