# Supplementary figures and images for: Pan- and core- gene association networks: Integrative approaches to understanding biological regulation
Source: PLoS One. 2019 Jan 9;14(1):e0210481. doi: 10.1371/journal.pone.0210481 (PMC6326509; doi:10.1371/journal.pone.0210481)

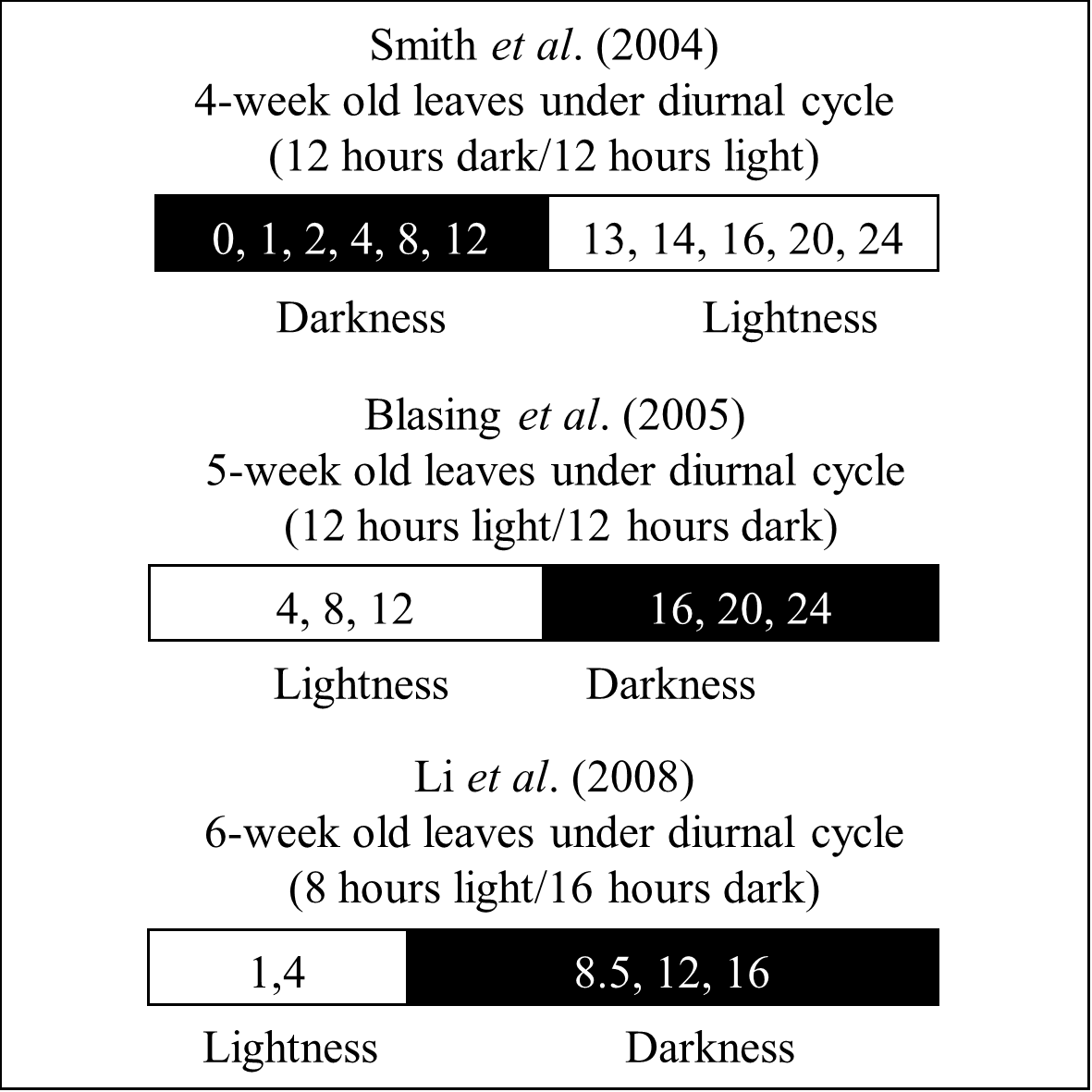

Supplement: S1 Fig — (1) Smith et al. (2004) collected the data at 1, 2, 4, 8 and 12 hours during the dark, and light periods (2) Blasing et al. (2005) collected the data at 4, 8 and 12 hours in both light/dark cycle conditions and (3) Li et al. (2008) collected the data at 1 and 4 hours during the light period and at 0.5, 4 and 8 hours during the dark period. (TIF) [file pone.0210481.s001.tif]

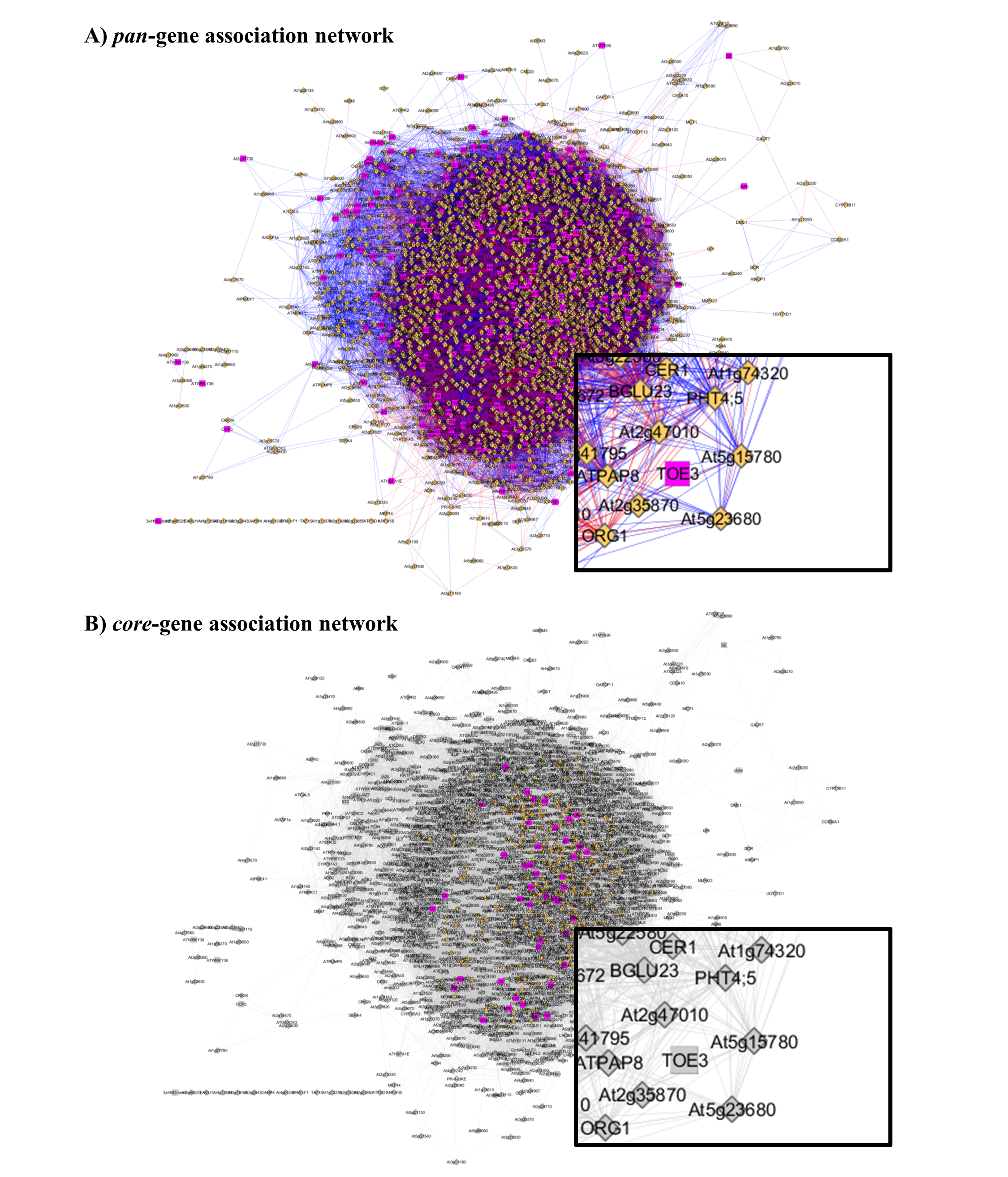

Supplement: S2 Fig — (A) pan-gene association network (pan-GAN) and (B) core-gene association network (core-GAN). The pink rectangles represent transcription factor genes, and the orange diamonds represent other genes (i.e., metabolic genes and signaling proteins). The gray symbols represent genes of pan-GAN that were absent in the core-GAN. The red and blue lines denote negative and positive correlation, respectively. (TIF) [file pone.0210481.s002.tif]

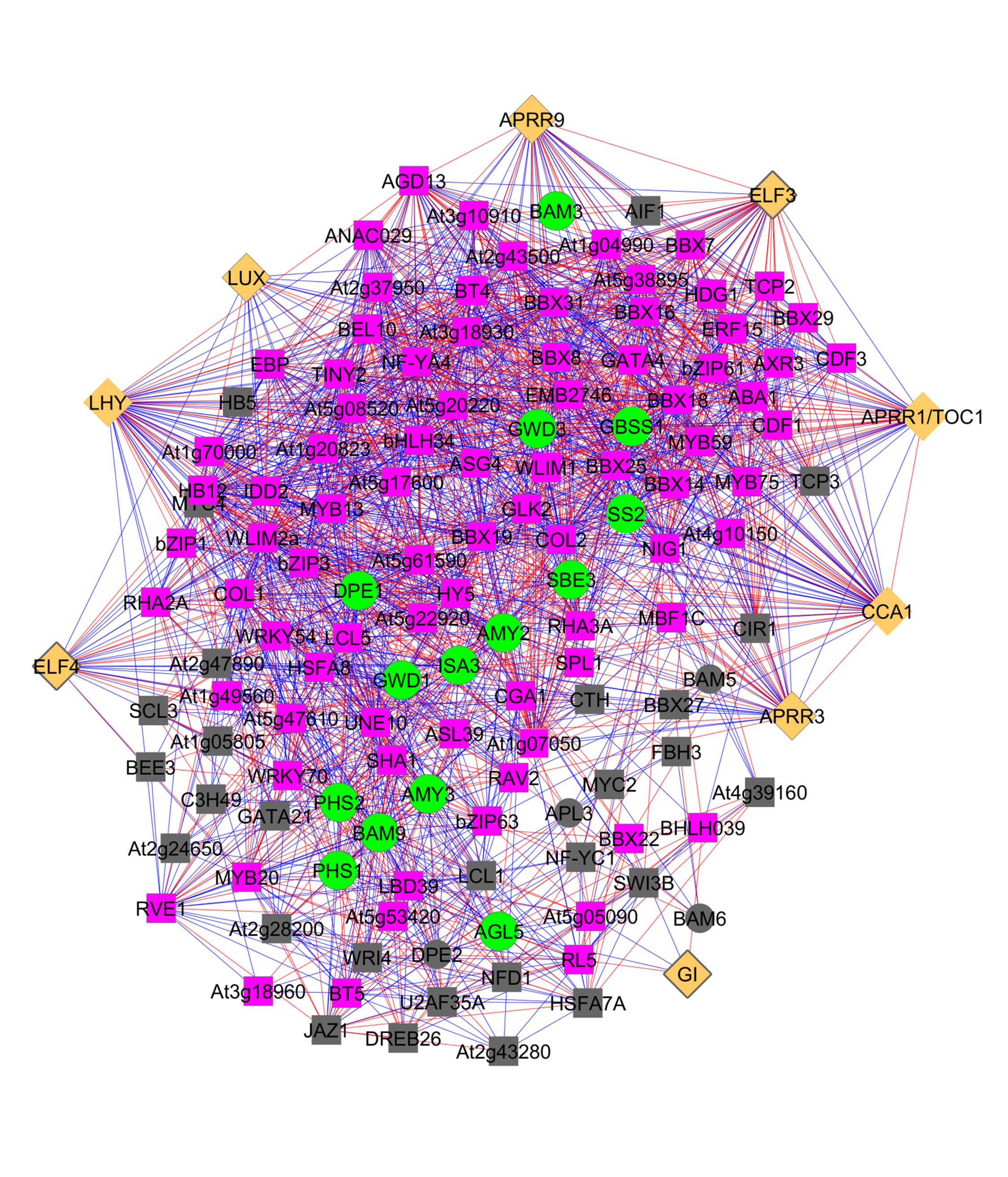

Supplement: S4 Fig — The orange diamonds represent circadian clock-related genes, the pink rectangles and the green circles represent TF and starch genes that are related to circadian clock genes. The gray symbols represent genes that are not correlated with circadian clock-related genes. The red and blue lines denote negative and positive correlation, respectively. (TIF) [file pone.0210481.s004.tif]
