# Supplementary material for: Pan- and core- gene association networks: Integrative approaches to understanding biological regulation
Source: PLoS One. 2019 Jan 9;14(1):e0210481. doi: 10.1371/journal.pone.0210481 (PMC6326509; doi:10.1371/journal.pone.0210481)
Supplement: S3 Fig — (A) cut-off varied according to the absolute magnitude of PCC values; (B) cut-off varied according to relative percentile rank of PCC values. (PDF) [file pone.0210481.s003.pdf]

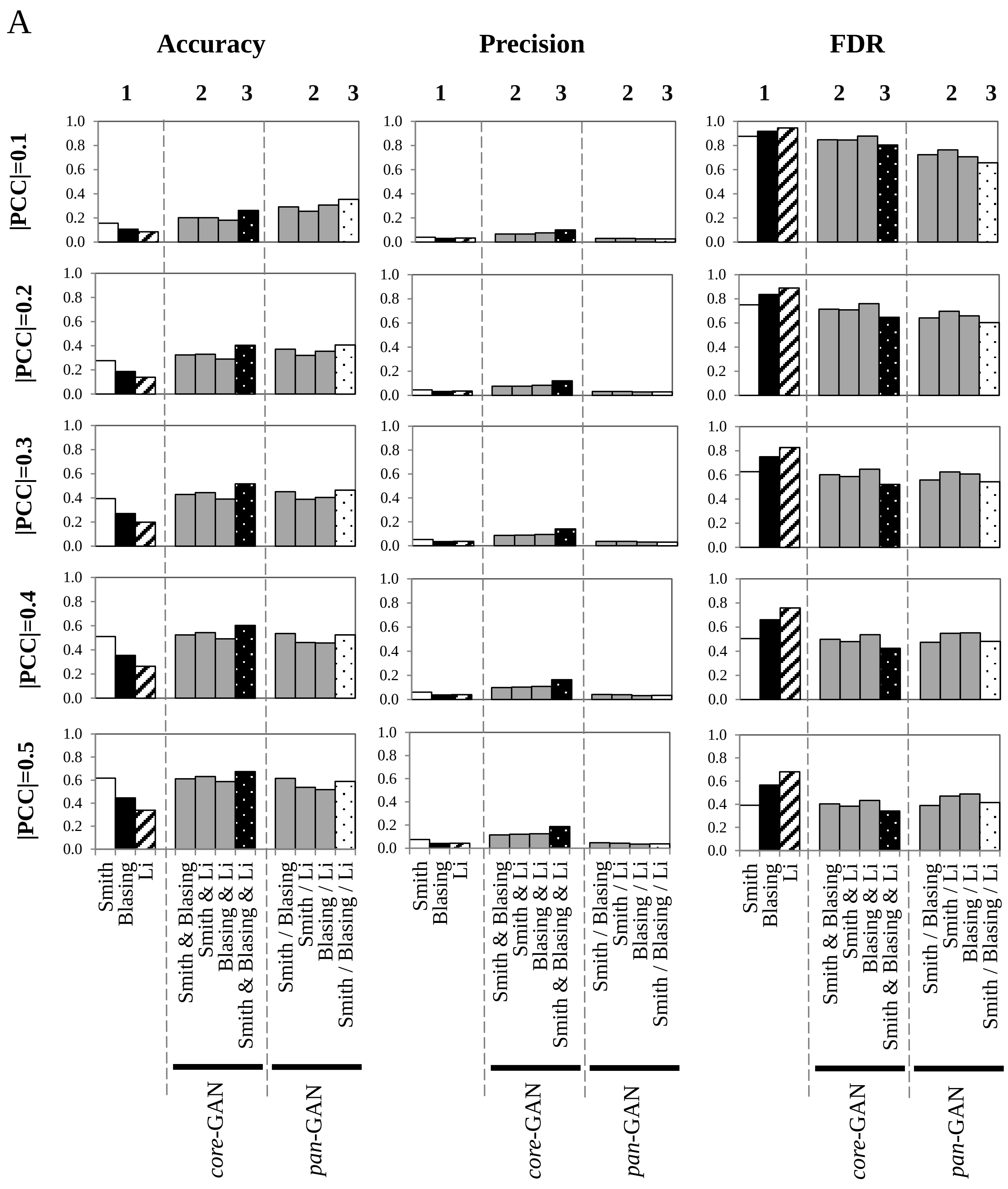
A
Accuracy
Precision
FDR
1
2
3
2
3
|PCC|=0.1
|PCC|=0.2
|PCC|=0.3
|PCC|=0.4
|PCC|=0.5
1.0
0.8
0.6
0.4
0.2
0.0
Smith
Blasing
Li
Smith & Blasing
Smith & Li
Blasing & Li
Smith & Blasing & Li
Smith / Blasing
Smith / Li
Blasing / Li
Smith / Blasing / Li
core-GAN
pan-GAN

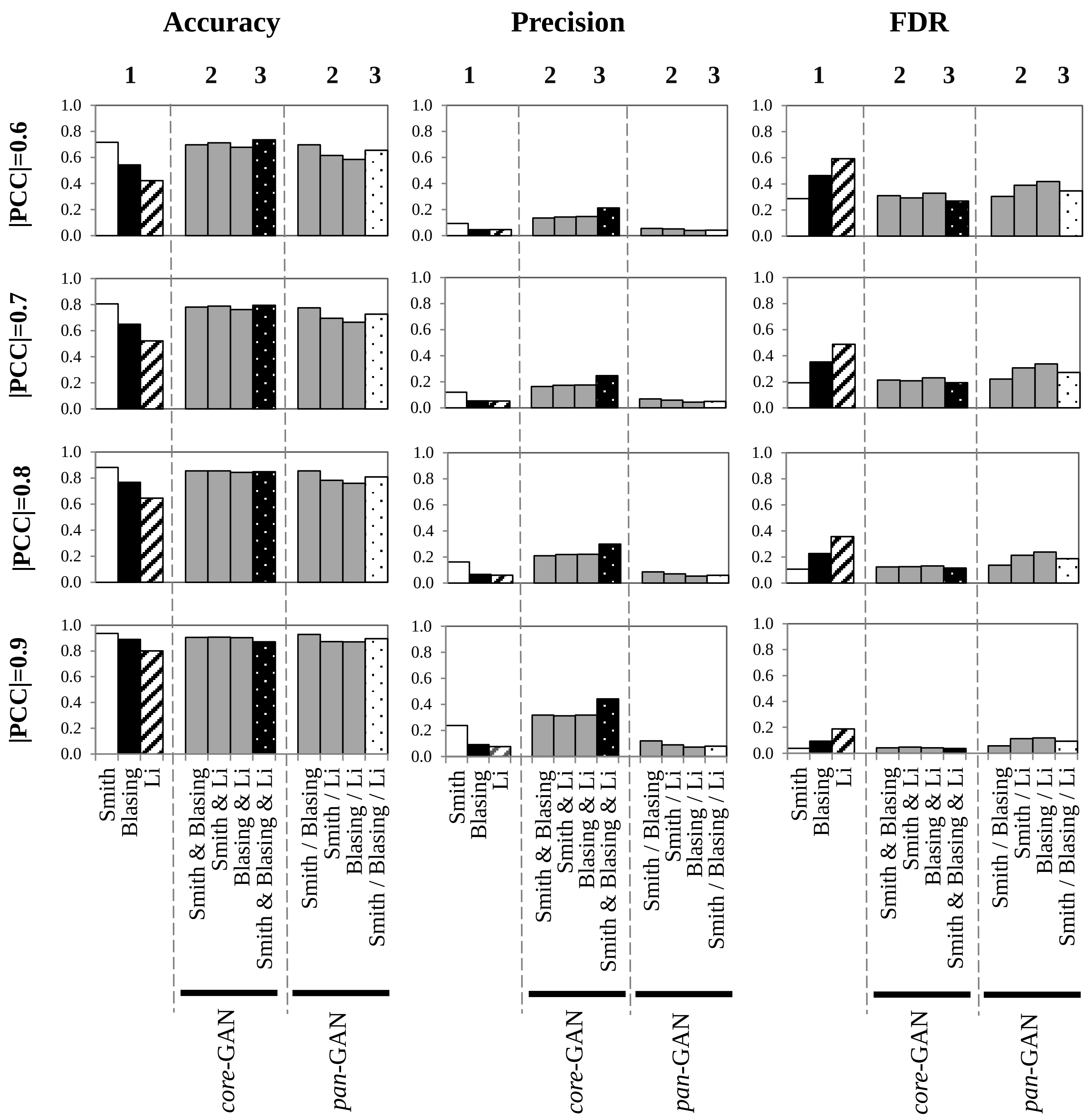
Accuracy
Precision
FDR
1
2
3
2
3
|PCC|=0.6
|PCC|=0.7
|PCC|=0.8
|PCC|=0.9
0.0
0.2
0.4
0.6
0.8
1.0
Smith
Blasing
Li
Smith & Blasing
Smith & Li
Blasing & Li
Smith & Blasing & Li
Smith / Blasing
Smith / Li
Blasing / Li
Smith / Blasing / Li
core-GAN
pan-GAN

B

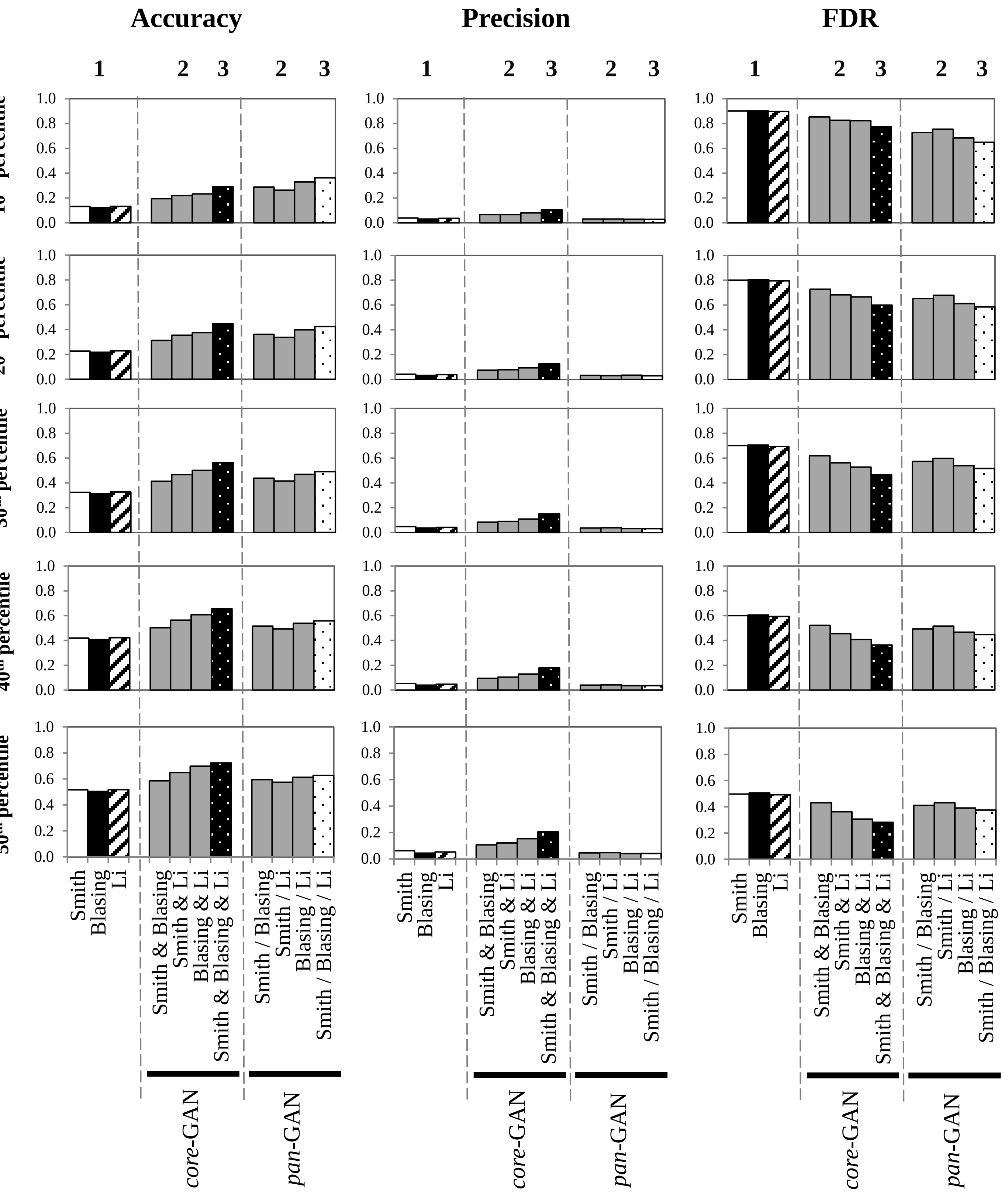
Accuracy
Precision
FDR
1
2
3
10th percentile
20th percentile
30th percentile
40th percentile
50th percentile
0.0
0.2
0.4
0.6
0.8
1.0
Smith
Blasing
Li
Smith & Blasing
Smith & Li
Blasing & Li
Smith & Blasing & Li
Smith / Blasing
Smith / Li
Blasing / Li
Smith / Blasing / Li
core-GAN
pan-GAN

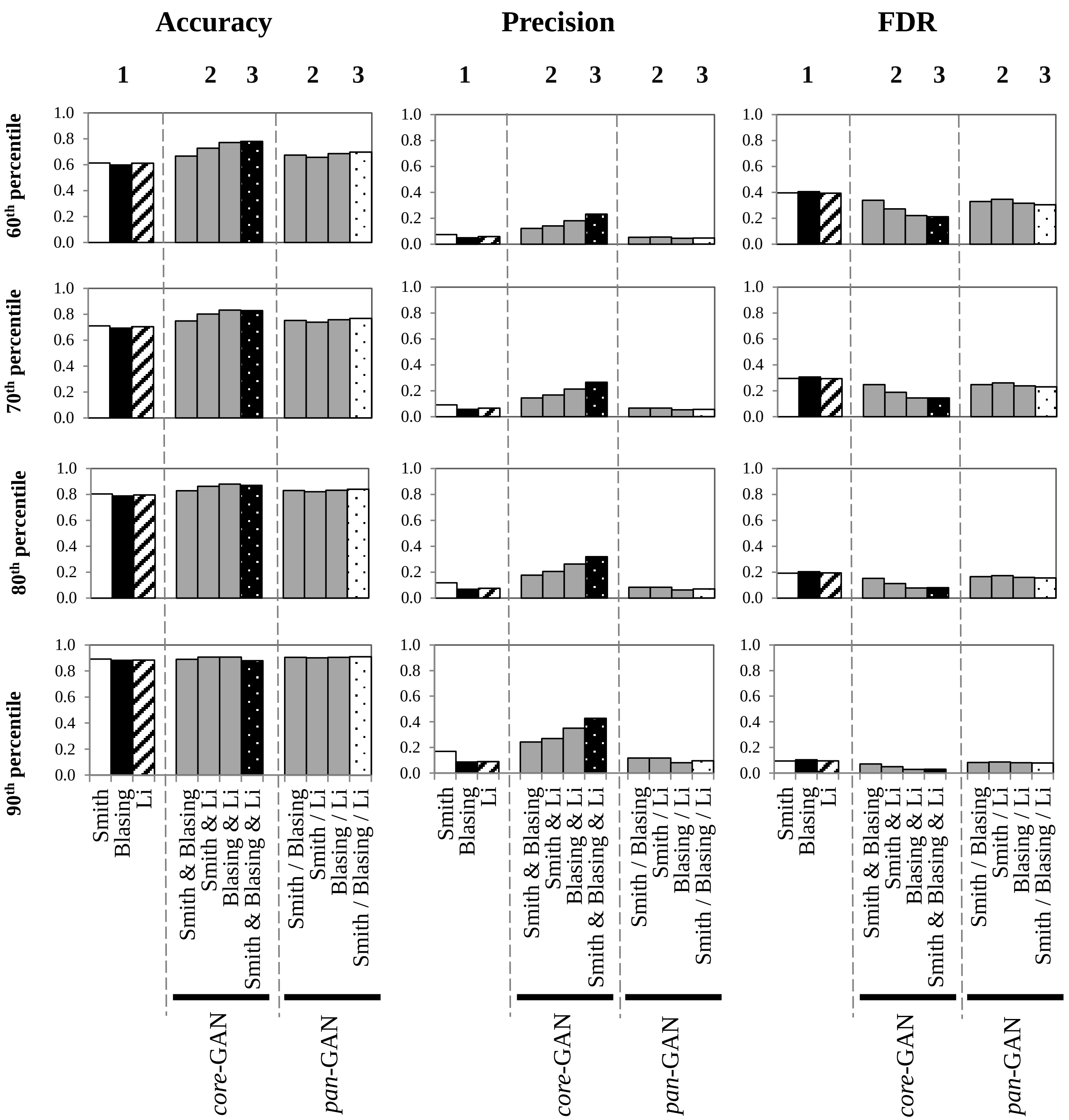
Accuracy
Precision
FDR
1
2
3
60th percentile
70th percentile
80th percentile
90th percentile
Smith
Blasing
Li
Smith & Blasing
Smith & Li
Blasing & Li
Smith & Blasing & Li
Smith / Blasing
Smith / Li
Blasing / Li
Smith / Blasing / Li
core-GAN
pan-GAN
