# Supplementary material for: Pan- and core- gene association networks: Integrative approaches to understanding biological regulation
Source: PLoS One. 2019 Jan 9;14(1):e0210481. doi: 10.1371/journal.pone.0210481 (PMC6326509; doi:10.1371/journal.pone.0210481)
Supplement: S1 Table — (PDF) [file pone.0210481.s005.pdf]

S1 Table. Top 10 TF genes with highest node degree (hub genes)

Smith-GAN			Blasing-GAN			Li-GAN		
**AGI**	**TF family**	**Node degree**	**AGI**	**TF family**	**Node degree**	**AGI**	**TF family**	**Node degree**
At1g10200	LIM	184	At5g02840	MYB_related	263	At3g18930	C3H	461
At2g46830	MYB_related	171	At1g66230	MYB	254	At3g02380	CO-like	457
At2g39900	LIM	163	At5g63780	C3H	253	At5g67480	TAZ	456
At1g01060	MYB_related	159	At4g00050	bHLH	250	At2g46830	MYB_related	450
At4g38960	DBB	151	At4g37540	LBD	245	At2g43500	Nin-like	449
At1g07050	C2C2-CO-like	145	At5g05090	G2-like	234	At2g21320	DBB	434
At2g31380	DBB	140	At1g19510	MYB_related	230	At3g21890	C2C2-CO-like	433
At2g21320	DBB	137	At5g15830	bZIP	227	At4g05330	C2H2	433
At3g21890	C2C2-CO-like	136	At5g28770	bZIP	198	At1g73870	CO-like	430
At5g65310	HD-ZIP	134	At4g30930	WRKY	197	At5g46830	bHLH	430
