# Supplementary material for: Pan- and core- gene association networks: Integrative approaches to understanding biological regulation
Source: PLoS One. 2019 Jan 9;14(1):e0210481. doi: 10.1371/journal.pone.0210481 (PMC6326509; doi:10.1371/journal.pone.0210481)
Supplement: S2 Table — (PDF) [file pone.0210481.s006.pdf]

S2 Table. Comparison of network performance among Smith-GAN, Blasing-GAN, Li-GAN, *core*- and *pan*-GAN by using co-expression network of 11,171 microarray dataset (ATTED database) as a reference network.

	Smith-GAN	Blasing-GAN	Li-GAN	*core*-GAN				*pan*-GAN			
				Smith & Blasing	Smith & Li	Blasing & Li	All	Smith / Blasing	Smith / Li	Blasing / Li	All
**All prediction**	23,001	54,327	123,895	6,318	8,967	5,408	2,909	71,010	137,929	172,815	183,440
**True positive**	4,873	3,975	7,803	1,819	2,556	1,508	1,171	7,029	10,120	10,270	11,939
**False positive**	15,680	39,872	95,504	3,908	5,648	3,246	1,481	51,644	105,536	132,130	139,735
**True negative**	395,759	388,949	411,092	88,893	112,242	76,094	37,862	861,479	828,993	983,250	1,349,225
**False negative**	11,963	8,534	9,377	5,508	6,310	5,057	4,336	16,244	16,542	15,155	20,741
**Accuracy**	0.935	0.890	0.800	0.906	0.906	0.903	0.870	0.928	0.873	0.871	0.895
**Precision**	0.237	0.091	0.076	0.318	0.312	0.312	0.442	0.120	0.088	0.072	0.079
**Sensitivity**	0.289	0.318	0.454	0.248	0.288	0.230	0.213	0.302	0.380	0.404	0.365
**Specificity**	0.962	0.907	0.811	0.958	0.952	0.959	0.962	0.943	0.887	0.882	0.906
**FPR***	0.038	0.093	0.189	0.042	0.048	0.048	0.038	0.057	0.113	0.118	0.094

* FPR is false positive rate
All is constructing TRN based on three transcriptome datasets
