# Supplementary material for: Pan- and core- gene association networks: Integrative approaches to understanding biological regulation
Source: PLoS One. 2019 Jan 9;14(1):e0210481. doi: 10.1371/journal.pone.0210481 (PMC6326509; doi:10.1371/journal.pone.0210481)
Supplement: S3 Table — (PDF) [file pone.0210481.s007.pdf]

S3 Table. Comparison of network performance among Smith-GAN, Blasing-GAN, Li-GAN, *core*- and *pan*-GAN by using co-expression network of 328 RNA-seq datasets (ATTED database) as a reference netwrok.

	Smith-GAN	Blasing-GAN	Li-GAN	*core*-GAN				*pan*-GAN			
				Smith & Blasing	Smith & Li	Blasing & Li	All	Smith / Blasing	Smith / Li	Blasing / Li	All
**All prediction**	23,001	54,327	123,895	6,318	8,967	5,408	2,909	71,010	137,929	172,815	183,440
**True positive**	4,159	3,917	5,970	1,741	2,090	1,389	1,079	6,335	8,039	8,498	9,905
**False positive**	16,492	41,584	97,702	3,974	6,165	3,513	1,600	54,102	108,029	135,773	143,726
**True negative**	397,685	391,914	415,102	93,435	115,785	81,680	40,843	847,237	826,592	970,884	1,321,918
**False negative**	9,939	7,681	8,077	5,046	5,246	4,369	3,756	13,729	14,376	13,598	18,307
**Accuracy**	0.938	0.889	0.799	0.913	0.912	0.913	0.887	0.926	0.872	0.868	0.892
**Precision**	0.201	0.086	0.058	0.305	0.253	0.283	0.403	0.105	0.069	0.059	0.064
**Sensitivity**	0.295	0.338	0.425	0.257	0.285	0.241	0.223	0.316	0.359	0.385	0.351
**Specificity**	0.960	0.904	0.809	0.959	0.949	0.959	0.962	0.940	0.884	0.877	0.902
**FPR***	0.040	0.096	0.191	0.041	0.051	0.041	0.038	0.060	0.116	0.123	0.098

* FPR is false positive rate

All is constructing TRN based on three transcriptome datasets
