# Supplementary material for: Pan- and core- gene association networks: Integrative approaches to understanding biological regulation
Source: PLoS One. 2019 Jan 9;14(1):e0210481. doi: 10.1371/journal.pone.0210481 (PMC6326509; doi:10.1371/journal.pone.0210481)
Supplement: S4 Table — (PDF) [file pone.0210481.s008.pdf]

S4 Table. Comparison of network performance among Smith-GAN, Blasing-GAN, Li-GAN, *core*- and *pan*-GAN by using transcriptional regulatory network based on direct TF-TG interactions in AtRegNet database as a reference network.

	Smith-GAN	Blasing-GAN	Li-GAN	*core*-GAN				*pan*-GAN			
				Smith & Blasing	Smith & Li	Blasing & Li	All	Smith / Blasing	Smith / Li	Blasing / Li	All
**All prediction**	23,001	54,327	123,895	6,318	8,967	5,408	2,909	71,010	137,929	172,815	183,440
**True positive**	2	14	15	0	0	0	0	16	17	29	31
**False positive**	9,008	20,160	48,372	2,697	3,472	2,259	1,226	26,471	53,908	66,273	70,338
**False negative**	279	248	272	151	159	147	96	357	370	360	418
**True negative**	198,401	170,231	185,611	49,478	60,989	40,959	21,898	377,706	377,690	414,028	565,969
**Accuracy**	0.955	0.893	0.792	0.946	0.944	0.945	0.943	0.934	0.874	0.861	0.889
**Precision**	0	0.001	0	0	0	0	0	0	0	0	0
**Sensitivity**	0.007	0.053	0.052	0	0	0	0	0.043	0.044	0.075	0.069
**Specificity**	0.957	0.894	0.793	0.948	0.946	0.948	0.947	0.935	0.875	0.862	0.889
**FPR***	0.043	0.106	0.207	0.052	0.054	0.052	0.053	0.065	0.125	0.138	0.111

* FPR is false positive rate

All is constructing TRN based on three transcriptome datasets
